# Supplementary material for: Stepwise Approach for Tracing the Geographical Origins of the Manila Clam Ruditapes philippinarum Using Dual-Element Isotopes and Carbon Isotopes of Fatty Acids
Source: Foods. 2022 Jul 1;11(13):1965. doi: 10.3390/foods11131965 (PMC9265916; doi:10.3390/foods11131965)
Supplement: Supplementary file 1 [file foods-11-01965-s001.zip › foods-1740373-supplementary.pdf]

<Supplementary Information>

Stepwise approach for tracing the geographical origins of the Manila clam *Ruditapes philippinarum* using dual-element isotopes and carbon isotopes of fatty acids

Young-Shin Go, Eun-Ji Won, Seung-Hee Kim, Dong-Hun Lee, Jung-Ha Kang, Kyung-Hoon Shin

Contents

Supplementary Text

- **Section S1**. Method for analyzing fatty acid content by GC-MS
- **Section S2**. Procedure for determining stable carbon isotope values of phospholipid fatty acids

Supplementary Figure

- **Figure S1.** Schematic of experimental procedure for analysis of fatty acid content and stable carbon isotope values (PLFA; phospholipid fatty acid, SFA; saturated fatty acid, USFA; unsaturated fatty acid, MUFA; monosaturated fatty acid, PUFA; polyunsaturated fatty acid).
- **Figure S2.** Concentration of fatty acid (μg/g dw) in clams collected in four different regions (13 sites) of Korea.

Supplementary Table

- **Table S1.** Fatty acid content of clams from Korea (each data indicates mean values from 3 individual clams, n = 51)
- **Table S2**. Number of individual clams with geographical origins determined by LDA of multi-factor combinations. Numbers indicates the samples whose origin was well identified out of the samples from each site (CC; n = 15, GN; n = 12, JB; n = 12, JN; n=12, total N=51). The second panel represents cross-validation results for each geographical origin using leave-one-out cross-validation (LOOCV) method.
- **Table S3.** The stable isotope ratios of carbon and nitrogen of Manila clams collected from three countries (China; n = 15, DPR Korea; n = 16, Korea; n = 102).

- **Table S4.** Content of fatty acids in the Manila clam *Ruditapes philippinarum* collected from three countries. P/S indicates ratio of PUFA to SFA. (China; n = 15, DPR Korea; n = 16, Korea; n = 51). Different letters indicate significant differences in isotope values according to country (ANOVA, Tukey's test, $P < 0.05$).
- **Table S5**. The carbon isotopic values for FAs of the Manila clam *Ruditapes philippinarum* collected from three countries (China; n = 3, DPR Korea; n = 3, Korea; n = 51). Different letters indicate significant differences in isotope values according to country (ANOVA, Tukey's test, P<0.05).
- **Table S6.** Number of individual clams with geographical origins defined by LDA of multi-factor combinations. Numbers indicates the samples whose origin was well identified out of the samples from each country (China; n = 3, DPR Korea; n = 3, Korea; n = 51). The second panel represents cross-validation results for each geographical origin using leave-one-out cross-validation (LOOCV) method.

Supplementary Text

Section S1. Method for analyzing fatty acid content by GC-MS

The total lipid extract (TLE) was used for fatty acid analysis. To extract neutral lipids, 1 mL of 1N KOH in MeOH was added and saponification was performed at 60°C for 1 h. After cooling, the neutral lipids were extracted with 1 mL of n-hexane. Neutral lipids were separated into two fractions on an Al_2O_3 column using n-hexane: DCM (9:1, v/v, for the apolar fraction) and DCM:MeOH (1:1, v/v, for the polar fraction). The polar FA fraction was reacted with 14% BF3 in MeOH at 60°C for 10 min. Methylated FAs were extracted thrice with 1 mL of DCM and 500 μL of distilled water. The biomarker FAs are phospholipids, and methylated FAs were separated into phospholipid FA (PLFA) fractions on the SiO_2 column using ethyl acetate solvent. The extracts were dried over anhydrous Na_2SO_4, dissolved in 100 μL of n-hexane and stored at −20°C until further analysis. The double bond positions in FAs were determined by dimethyl disulfide (DMDS) analysis, according to a previous study [Nichols et al., 1986]. For DMDS analysis, aliquots of PLFA samples (100 μL) were treated with 20 μL of iodine solution (6%, w/v in diethyl ether). The formation of DMDS adducts was performed at 50°C for 48 h. After cooling, 500 μL of n-hexane was added, and iodine was removed with 500 μL of sodium thiosulfate solution (5%, w/v in water). The combined organic layers were concentrated under a gentle stream of nitrogen and dissolved in 50 μL of n-hexane prior to further analysis. The concentrations of PLFA were determined by GC analysis using a DB-5 silica capillary column (60 m × 0.25 mm, 0.25 μm, Agilent) in an Agilent 7890 Series in splitless mode with helium as the carrier gas. The initial GC oven temperature was set to 60°C, which was subsequently increased to 130°C at a rate of 20°C /min, raised to 320°C at a rate of 4°C /min, and maintained for 40 min. The injector and detector temperatures were set at 290°C and 300°C. The concentration of each PLFA compound was calculated relative to the internal standard (Supelco 37 Component FAME Mix, Supelco, and Bellefonte, PA, USA). The PLFA was identified by gas chromatography mass spectrometry (GC-MS) using an Agilent GC interfaced to 5977 B MS. The GC-MS was operated in electron impact (EI) mode at 70 eV with a full scan mass range of m/z 50-800 (cycle time of 0.9 s, resolution of 1000). GC-MS was performed using a HP-5MS column (25 m × 0.25 mm, 0.10 μm, Agilent). Helium was used as the carrier gas (1.2 mL/min). The samples were injected in splitless mode and subjected to the same temperature program used for the GC analysis. The molecular structures were determined by comparing their mass spectral fragmentation patterns and retention times with previously published data.

Reference

Nichols, P.D., Guckert, J.B., White, D.C., 1986, Determination of monounsaturated fatty acid double-bond position and geometry for microbial monocultures and complex consortia by capillary GC-MS of their dimethyl disulphide adducts. J. Microbiol. Methods 5, 49-55.

Section S2. Procedure for determining stable carbon isotope values of phospholipid fatty acids

The stable carbon isotope values of the PLFA were determined using GC-C-IRMS. An isotope ratio mass spectrometer (isoprime visION, Elementar) was coupled to a gas chromatograph (Hewlett Packard 7890 N series, Agilent Technologies, Santa Clara, CA, USA) via a combustion interface (glass tube packed with copper oxide (CuO), operated at 850°C). The samples were subjected to the same temperature conditions and capillary column as those described for the GC-MS analysis. Calibration was performed by injecting multiple pulses of the reference gas CO_2 of known stable carbon isotopic values at the beginning and end of each sample run. Isotopic values are expressed as δ notation in per mil (‰) relative to Vienna Pee Dee Belemnite (VPDB). The correlation coefficients (R^2) of the known stable carbon isotopic values of the certified isotope standards and the average values of the measured samples were higher than 0.99. In the case of PLFA, the stable carbon isotopic value measurements needed to be corrected for the additional carbon atoms for derivatization with BF3/MeOH. To monitor the accuracy of the measurements, standards with known stable carbon isotopic values were repeatedly analyzed every five sample runs. The stable carbon isotopic values of PLFA are expressed as follows:

$$\delta^{13}C_B = [(n + 1) \times \delta^{13}C_A - \delta^{13}C_{MeOH}]/n \quad (1)$$

Eq.1 The formula to correct the methylation effect on stable carbon isotopes of FAs. $\delta^{13}C_A$ is the measured stable carbon isotopic value of the PLFA, and $\delta^{13}C_{MeOH}$ is the stable carbon isotopic value of methanol. $\delta^{13}C_B$ is the corrected stable carbon isotopic value of PLFA, which does not include carbon from methanol, and n is the number of carbon atoms in non-methylated FA.

Figure S1. Schematic of experimental procedure for analysis of the fatty acid content and stable carbon isotope values (PLFA; phospholipid fatty acid, SFA; saturated fatty acid, USFA; unsaturated fatty acid, MUFA; monosaturated fatty acid, PUFA; polyunsaturated fatty acid).

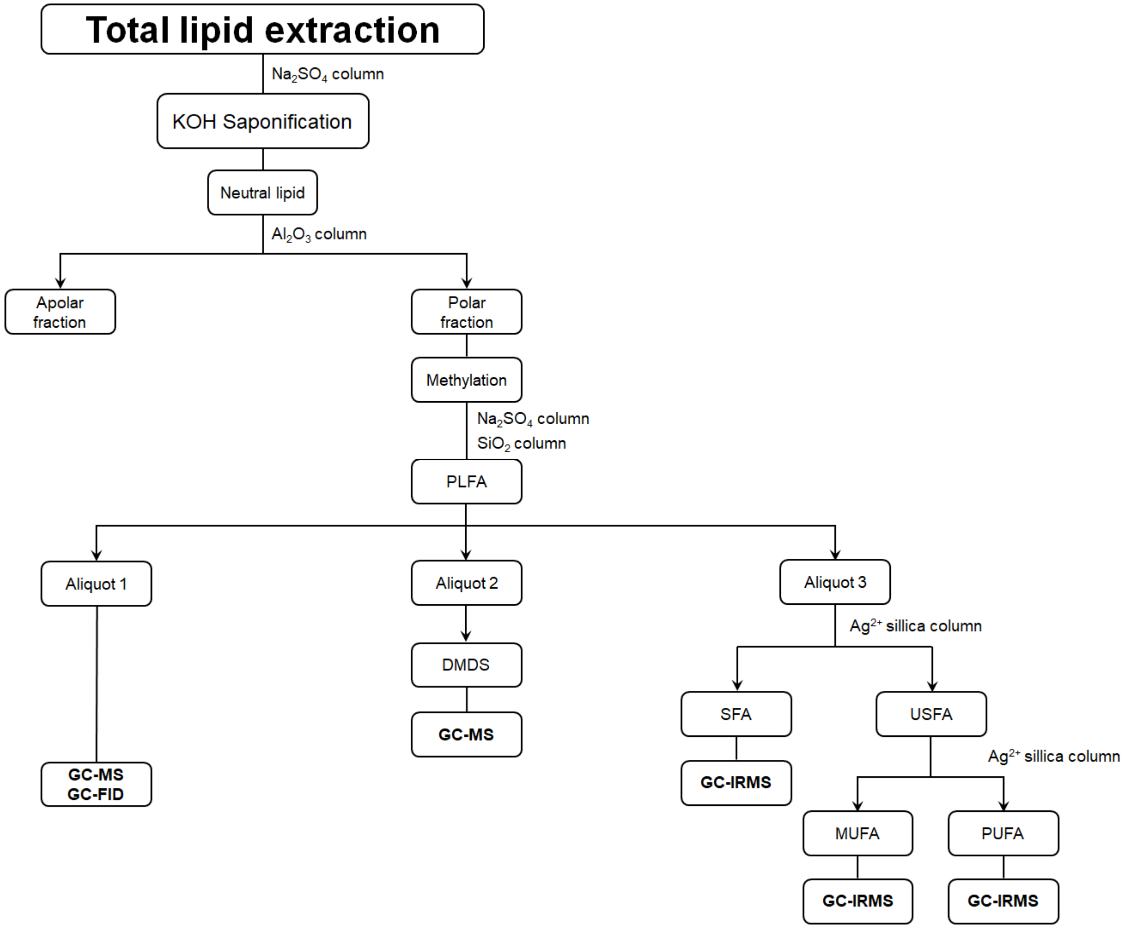

Fig. S2. Concentration of fatty acid (μg/g dw) in clams collected from four different regions (13 sites) in Korea.

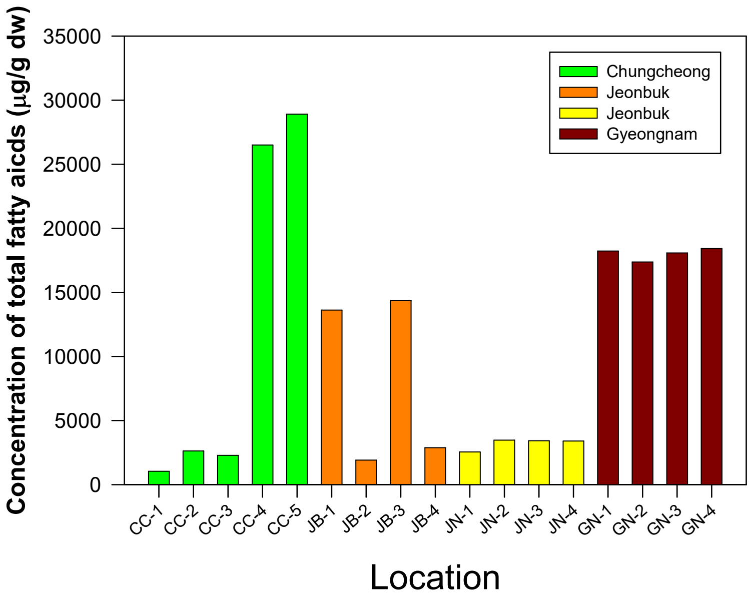

Table S1. Fatty acid content (%) of clams from Korea (each data indicates mean values from 3 individual clams, n = 51).

**Fatty acid (%)**	**CC-1**	**CC-2**	**CC-3**	**CC-4**	**CC-5**	**JB-1**	**JB-2**	**JB-3**	**JB-4**	**JN-1**	**JN-2**	**JN-3**	**JN-4**	**GN-1**	**GN-2**	**GN-3**	**GN-4**
	Dangjin	Sogeunri	Uihang	Hongseong	Boryeong	Saemangeum	Wedo	Gochang	Shinan	Wando	Hwayang	Dolsan	Yeosu	Tongyeong	Geoje	Masan	Udo
14:0	1.03±0.09	2.15±0.09	2.22±0.12	0.72±0.05	0.72±0.05	0.68±0.03	1.03±0.05	0.85±0.13	1.42±0.19	1.83±0.05	0.84±0.04	0.77±0.05	0.83±0.07	1.11±0.07	1.14±0.25	0.77±0.08	0.83±0.06
15:0	1.78±0.08	0.81±0.05	0.84±0.06	0.71±0.03	0.77±0.05	0.78±0.03	0.86±0.11	0.77±0.05	0.90±0.05	0.69±0.01	0.65±0.01	0.65±0.04	0.59±0.05	0.81±0.18	0.86±0.07	0.83±0.06	0.82±0.08
16:0*	15.68±0.65	45.35±0.90	40.04±1.46	32.36±1.50	30.56±0.57	26.25±0.58	27.17±0.54	25.89±0.28	37.74±1.01	29.20±0.77	29.93±0.15	30.29±0.20	30.99±0.42	28.39±0.71	31.28±0.73	29.74±0.55	29.00±0.95
17:0	1.15±0.12	1.76±0.11	2.05±0.15	2.99±0.02	2.48±0.32	2.48±0.10	2.24±0.21	2.29±0.10	2.17±0.18	2.27±0.19	2.91±0.07	2.93±0.06	2.80±0.07	2.49±0.28	3.05±0.07	2.92±0.13	2.57±0.36
18:0*	6.58±0.26	15.41±0.68	13.26±0.26	16.18±0.72	15.94±0.31	14.12±0.23	13.87±0.32	14.13±0.15	14.83±0.47	11.93±0.34	18.08±0.19	18.25±0.15	18.25±0.10	18.16±0.58	18.36±0.24	19.38±0.53	18.57±0.57
**SFAs**	**26.20±0.93**	**65.48±1.74**	**58.40±1.16**	**52.96±0.83**	**50.46±1.04**	**44.32±0.40**	**45.17±0.31**	**43.93±0.32**	**43.93±0.75**	**45.91±0.52**	**52.41±0.11**	**52.89±0.06**	**53.46±0.45**	**50.96±1.20**	**54.68±1.02**	**53.64±0.76**	**51.79±1.39**
16:1n-7	7.02±0.09	5.83±0.87	6.26±0.29	3.05±0.07	3.06±0.11	3.20±0.07	3.66±0.09	3.80±0.40	4.19±0.18	5.99±0.10	2.78±0.03	2.82±0.05	2.82±0.08	6.38±0.52	5.85±0.24	6.56±0.33	7.53±0.27
18:1n-7*	5.53±0.26	5.42±0.42	6.10±0.13	3.48±0.33	4.44±0.08	6.93±0.14	4.45±0.28	6.98±0.09	5.40±0.26	5.53±0.16	4.56±0.05	4.67±0.17	4.44±0.10	9.52±0.45	7.75±0.28	7.36±0.43	7.83±0.08
18:1n-9*	5.54±0.29	4.15±0.24	4.43±0.12	5.44±0.27	5.35±0.10	7.21±0.11	5.94±0.06	7.09±0.07	3.15±0.24	3.28±0.07	3.49±0.33	3.53±0.25	3.53±0.14	5.76±0.40	5.29±0.14	5.35±0.12	5.32±0.30
20:1n-7*	5.43±0.29	3.92±0.53	5.09±0.34	5.03±0.15	4.76±0.10	6.93±0.05	4.35±0.21	7.00±0.03	4.46±0.25	4.87±0.15	4.43±0.19	4.52±0.03	4.39±0.25	6.15±0.26	5.84±0.09	6.32±0.17	6.30±0.28
20:1n-9*	15.86±0.31	4.66±0.78	4.52±0.08	5.07±0.17	4.66±0.02	6.91±0.04	13.97±0.17	6.74±0.12	12.91±0.78	11.56±0.45	10.93±0.40	10.35±0.41	9.56±0.46	3.53±0.42	3.19±0.31	3.05±0.06	3.03±0.07
**MUFAs**	**39.38±0.68**	**23.98±1.52**	**26.41±0.52**	**22.07±0.37**	**22.27±0.20**	**31.19±0.33**	**32.37±0.69**	**31.60±0.29**	**31.60±0.40**	**31.23±0.30**	**26.18±0.09**	**25.90±0.74**	**24.74±0.26**	**31.34±0.92**	**27.92±0.45**	**28.64±0.47**	**30.01±0.44**
18:2n-6	1.16±0.18	0.56±0.10	0.95±0.09	0.33±0.06	0.39±0.02	0.46±0.02	0.70±0.05	0.51±0.04	1.57±0.27	0.99±0.02	0.96±0.07	0.81±0.04	0.96±0.11	0.58±0.08	0.63±0.14	0.51±0.04	0.51±0.04
20:4n-6	6.91±0.27	1.06±0.07	2.12±0.23	0.90±0.09	0.84±0.05	0.98±0.04	3.30±0.19	0.92±0.04	2.45±0.25	2.95±0.09	3.97±0.10	4.09±0.03	3.71±0.46	0.88±0.10	0.78±0.03	0.84±0.09	0.80±0.06
20:5n-3	15.80±0.30	6.79±0.25	6.12±0.51	13.94±0.36	16.08±0.55	14.94±0.51	9.18±0.43	15.75±0.77	2.47±0.23	9.83±0.18	7.70±0.08	7.88±0.07	8.40±0.50	7.27±1.09	6.94±0.27	7.04±0.52	6.91±0.32
21:5n-3	2.24±0.06	0.50±0.07	1.21±0.38	0.41±0.08	0.37±0.05	0.39±0.02	1.16±0.19	0.42±0.04	1.37±0.03	1.25±0.06	1.37±0.08	1.42±0.04	1.25±0.13	0.17±0.05	0.17±0.03	0.18±0.07	0.31±0.03
22:6n-3	8.30±0.47	1.62±0.35	4.79±0.60	9.41±0.45	9.59±0.34	7.73±0.95	8.12±0.95	6.87±0.88	4.97±0.43	7.83±0.37	7.40±0.14	7.01±0.65	7.49±0.33	8.80±1.12	8.89±0.81	9.15±0.56	9.67±0.71
**PUFAs**	**34.41±0.38**	**10.54±0.57**	**15.19±1.66**	**24.98±0.85**	**27.26±0.95**	**24.49±0.73**	**22.46±0.89**	**24.47±0.07**	**24.47±0.61**	**22.86±0.25**	**21.41±0.08**	**21.21±0.69**	**21.81±0.61**	**17.70±2.05**	**17.40±0.59**	**17.72±0.72**	**18.20±1.01**
**P/S**	**1.31±0.06**	**0.16±0.01**	**0.26±0.03**	**0.47±0.02**	**0.54±0.03**	**0.55±0.02**	**0.50±0.02**	**0.56±0.01**	**0.56±0.01**	**0.50±0.01**	**0.41±0.01**	**0.40±0.01**	**0.41±0.01**	**0.35±0.05**	**0.32±0.02**	**0.33±0.02**	**0.35±0.03**
**DHA/EPA**	**0.52±0.02**	**0.24±0.04**	**0.78±0.04**	**0.67±0.02**	**0.60±0.01**	**0.52±0.08**	**0.89±0.13**	**0.44±0.08**	**0.44±0.22**	**0.80±0.05**	**0.96±0.03**	**0.89±0.07**	**0.89±0.04**	**1.22±0.08**	**1.28±0.61**	**1.30±0.12**	**1.40±0.07**

* Asterisks indicate fatty acids whose isotopic ratios were analyzed using GC-IRMS. Because of the measurement conditions, including the peak height and separation state, it was not possible to determine $\delta^{13}C$ values for all fatty acids. The procedures for fatty acids and their analytical conditions in GC are described in Text S1 and S2.

Table S2. Number of individual clams with geographical origins defined by LDA of multi-factor combinations. Numbers indicates the samples whose origin was well identified out of samples from each site (CC; n = 15, GN; n = 12, JB; n = 12, JN; n=12, total N=51). The second panel represents cross-validation results of each geographical origin using leave-one-out cross-validation (LOOCV) method.

Regions	Number of correctly classified clams			
	**SIA + FAs**	**FAs +δ^{13}C-FAs**	**SIA + δ^{13}C-FAs**	**All methods**
**Chungcheong (CC)**	15	15	15	15
**Gyeongnam (GN)**	12	12	12	12
**Jeonbuk (JB)**	12	12	11	12
**Jeonnam (JN)**	12	12	12	12
**Total**	**51 (100%)**	**51 (100%)**	**50 (98.03%)**	**51 (100%)**
	Cross-validated value			
**Chungcheong**	15	15	15	15
**Gyeongnam**	12	12	12	12
**Jeonbuk**	12	12	11	12
**Jeonnam**	12	12	12	12
**Total**	**51 (100%)**	**51 (100%)**	**50 (98.03%)**	**51 (100%)**

Table S3. The stable isotope ratios of carbon and nitrogen of Manila clams collected in three countries (China; n = 15, DPR Korea; n = 16, Korea; n = 102).

Country	Location	ID	δ13C (‰)	δ15N (‰)
China	Dalian	CHN	-17.5	8.5
			-17.5	8.5
			-17.6	8.7
			-18.2	9.0
			-18.1	9.0
			-17.7	9.9
			-16.9	9.7
			-17.4	10.2
			-18.1	10.2
			-17.9	10.7
			-17.2	10.5
			-17.3	10.1
			-17.2	10.1
			-17.2	10.3
			-17.8	9.8
		**Max.**	**-16.9**	**10.7**
		**Min.**	**-18.2**	**8.57**
		**Mean.**	**-17.6**	**9.7**
		**S.D.**	**0.4**	**0.7**
DPR Korea	-	NK	-17.1	9.1
			-17.1	8.7
			-17.7	8.9
			-17.9	9.2
			-17.8	9.3
			-17.5	8.9
			-17.1	9.1
			-17.3	8.7
			-17.7	8.9
			-17.4	9.3
			-17.8	8.7
			-18.2	9.1
			-17.8	8.5
			-18.3	8.8
			-17.8	8.99
			-18.2	9.10
		**Max.**	**-17.08**	**9.3**
		**Min.**	**-18.31**	**8.5**
		**Mean.**	**-17.69**	**9.0**
		**S.D.**	**0.38**	**0.2**
Korea	Dangjin	CC-1	-18.1	8.8
			-18.1	9.1
			-17.9	8.8
			-18.3	8.7
			-17.7	9.1
		**Max.**	**-17.7**	**9.1**
		**Min.**	**-18.3**	**8.7**
		**Mean.**	**-18.0**	**8.9**
		**S.D.**	**0.2**	**0.2**
	Sogeunri	CC-2	-18.3	6.0
			-18.4	6.5
			-18.6	6.6
			-18.3	5.9
			-18.4	6.9
		**Max.**	**-18.3**	**6.9**
		**Min.**	**-18.6**	**5.9**
		**Mean.**	**-18.4**	**6.4**
		**S.D.**	**0.1**	**0.4**

Table S3. (Continued).

Country	Location	ID	$\delta^{13}C$ (‰)	$\delta^{15}N$ (‰)
Korea	Uihang	CC-3	-17.9	7.3
			-18.6	6.7
			-18.7	6.9
			-18.1	6.7
			-18.4	6.4
		**Max.**	**-17.9**	**7.3**
		**Min.**	**-18.7**	**6.4**
		**Mean.**	**-18.3**	**6.8**
		**S.D.**	**0.3**	**0.3**
	Hongseong	CC-4	-17.1	9.2
			-17.5	9.2
			-17.8	9.0
			-18.0	9.1
			-17.8	8.8
		**Max.**	**-17.1**	**9.2**
		**Min.**	**-18.0**	**8.8**
		**Mean.**	**-17.6**	**9.1**
		**S.D.**	**0.4**	**0.2**
	Boryeong	CC-5	-17.3	8.1
			-17.6	8.8
			-17.2	9.0
			-17.7	8.4
			-17.8	8.7
		**Max.**	**-17.2**	**9.0**
		**Min.**	**-17.9**	**8.1**
		**Mean.**	**-17.5**	**8.6**
		**S.D.**	**0.3**	**0.4**
	Saemangeum	JB-1	-16.9	9.9
			-16.9	10.4
			-17.3	9.1
			-17.3	9.6
			-17.1	9.4
			-17.3	9.0
			-17.1	9.7
			-17.1	9.7
			-17.1	10.0
			-17.2	9.4
		**Max.**	**-16.9**	**10.4**
		**Min.**	**-17.3**	**9.0**
		**Mean.**	**-17.1**	**9.6**
		**S.D.**	**0.1**	**0.4**
	Wedo	JB-2	-16.3	9.6
			-16.7	9.0
			-16.6	8.9
			-16.7	9.0
			-17.0	8.8
		**Max.**	**-16.3**	**9.6**
		**Min.**	**-17.0**	**8.8**
		**Mean.**	**-16.6**	**9.1**
		**S.D.**	**0.3**	**0.3**
	Gochang	JB-3	-17.4	10.2
			-17.3	10.1
			-17.6	10.2
			-17.2	10.0
			-17.3	9.6
		**Max.**	**-17.2**	**10.2**
		**Min.**	**-17.6**	**9.6**
		**Mean.**	**-17.4**	**10.0**
		**S.D.**	**0.1**	**0.3**

Table S3. (Continued).

Country	Location	ID	δ¹³C (‰)	δ¹⁵N (‰)
Korea	Shinan	JB-4	-15.9	8.9
			-16.6	8.9
			-16.3	8.8
			-17.0	8.7
			-16.4	8.0
		**Max.**	**-15.9**	**8.9**
		**Min.**	**-17.0**	**8.0**
		**Mean.**	**-16.4**	**8.7**
		**S.D.**	**0.4**	**0.4**
	Wando	JN-1	-16.2	7.6
			-16.5	7.6
			-16.4	7.7
			-16.6	7.6
			-15.9	7.7
			-16.6	7.7
			-16.7	7.9
			-16.7	7.8
			-16.9	7.7
		**Max.**	**-15.9**	**7.9**
		**Min.**	**-16.9**	**7.6**
		**Mean.**	**-16.5**	**7.7**
		**S.D.**	**0.3**	**0.1**
	Hwayang	JN-2	-17.0	9.1
			-17.0	8.9
			-16.6	9.1
		**Max.**	**-16.6**	**9.1**
		**Min.**	**-17.0**	**8.9**
		**Mean.**	**-16.9**	**9.0**
		**S.D.**	**0.2**	**0.1**
	Dolsan	JN-3	-17.0	9.1
			-17.0	8.9
			-16.6	9.1
		**Max.**	**-16.6**	**9.1**
		**Min.**	**-17.0**	**8.9**
		**Mean.**	**-16.9**	**9.0**
		**S.D.**	**0.2**	**0.1**
	Yeosu	JN-4	-16.5	9.2
			-16.6	9.2
			-17.1	8.9
			-17.1	9.0
			-17.0	9.0
			-16.8	9.1
			-17.0	9.2
			-16.9	9.0
			-16.9	9.2
			-17.0	9.1
		**Max.**	**-16.5**	**9.2**
		**Min.**	**-17.1**	**8.9**
		**Mean.**	**-16.9**	**9.1**
		**S.D.**	**0.2**	**0.1**
	Tongyeong	GN-1	-16.3	9.1
			-16.5	8.6
			-17.2	9.0
			-16.8	8.5
			-17.0	8.9
			-16.9	8.7
			-17.0	8.8
			-16.9	8.7
		**Max.**	**-16.3**	**9.1**
		**Min.**	**-17.2**	**8.5**
		**Mean.**	**-16.8**	**8.8**
		**S.D.**	**0.3**	**0.2**

Table S3. (Continued).

Country	Location	ID	δ13C (‰)	δ15N (‰)
Korea	Geoje	GN-2	-15.7	10.2
			-15.5	10.5
			-15.7	10.6
			-15.6	10.8
			-15.6	10.7
			-15.7	10.6
			-15.6	10.5
			-15.9	10.4
			-15.8	10.5
			-15.5	10.6
		**Max.**	**-15.5**	**10.8**
		**Min.**	**-15.9**	**10.2**
		**Mean.**	**-15.7**	**10.6**
		**S.D.**	**0.1**	**0.2**
	Masan	GN-3	-16.5	10.0
			-16.3	9.8
			-16.2	9.7
		**Max.**	**-16.2**	**10.0**
		**Min.**	**-16.5**	**9.7**
		**Mean.**	**-16.3**	**9.8**
		**S.D.**	**0.1**	**0.12**
	Udo	GN-4	-15.6	11.1
			-15.8	11.0
			-15.9	10.9
			-16.1	11.2
			-15.8	10.9
		**Max.**	**-15.6**	**11.2**
		**Min.**	**-16.1**	**10.9**
		**Mean.**	**-15.8**	**11.0**
		**S.D.**	**0.2**	**0.1**

Table S4. Content of fatty acids in the Manila clam *Ruditapes philippinarum* collected from three countries. P/S indicates ratio of PUFA to SFA. (China; n = 15, DPR Korea; n = 16, Korea; n = 51). Different letters indicate significant differences in isotope values according to countries (ANOVA, Tukey's, $P < 0.05$).

	Saturated Fatty Acids (%)					Σ	Ratio
	**C14:0**	**C15:0**	**C16:0**	**C17:0**	**C18:0**	**SFAs**	P/S
**China**	1.12±0.23 [a]	0.88±0.34 [a]	26.02±1.85 [a]	2.53±0.29 [a]	20.17±1.03 [b]	50.73±0.75	0.48±0.03
**DPR Korea**	1.04±0.07 [a]	2.09±2.53 [b]	27.65±1.42 [a]	2.19±0.23 [a]	10.42±1.08 [a]	43.39±1.07	0.48±0.02
**Korea**	1.11±0.49 [a]	0.83±0.26 [a]	30.58±6.27 [a]	2.44±0.51 [a]	15.60±3.18 [b]	50.57±2.14	0.45±0.03
	**Monounsaturated Fatty Acids (%)**						
	**C16:1n-7**	**C 18:1n-7**	**C 18:1n-9**	**C 20:1n-7**	**C 20:1n-9**	**MUFAs**	DHA/EPA
**China**	4.21±0.18 [a]	7.31±0.44 [a]	5.07±0.47 [a]	4.18±0.44 [a]	4.21±0.21 [a]	24.98±0.35 [a]	0.29±0.12
**DPR Korea**	9.23±0.25 [b]	11.42±0.24 [b]	4.47±0.67 [a]	5.66±0.82 [a]	5.02±0.04 [a]	35.80±0.40 [b]	0.59±0.07
**Korea**	4.75±1.70 [a]	5.91±1.58 [a]	4.93±1.25 [a]	5.28±0.96 [a]	7.68±4.16 [a]	28.55±1.93 [a]	0.91±0.09
	**Polyunsaturated Fatty Acids (%)**						
	**C 18:2n-6**	**C 20:4n-6**	**C 20:5n-3**	**C 21:5n-3**	**C 22:6n-3**	**PUFAs**	
**China**	0.31±0.03 [a]	0.82±0.04 [a]	17.71±0.82 [b]	0.37±0.04 [a]	5.07±1.81 [a]	24.28±0.55	
**DPR Korea**	0.53±0.05 [a]	0.83±0.04 [a]	12.12±0.84 [ab]	0.18±0.03 [a]	7.16±0.28 [a]	20.81±0.25	
**Korea**	0.74±0.33 [a]	2.20±1.71 [a]	9.59±4.05 [a]	0.83±0.60 [a]	7.51±2.11 [a]	20.88±1.76	

Table S5. The carbon isotopic values for FAs of the Manila clam *Ruditapes philippinarum* collected from three countries (China; n = 3, DPR Korea; n = 3, Korea; n = 51). Different letters indicate significant differences in isotope values according to countries (ANOVA, Tukey's, P<0.05).

Country	δ^{13}C 16:0	δ^{13}C 18:0	δ^{13}C 18:1n-7	δ^{13}C 18:1n-9	δ^{13}C 20:1n-7	δ^{13}C 20:1n-9
China	−25.2±0.5 ^{a}	−22.0±0.6 ^{a}	−16.5±0.4 ^{a}	−19.45±0.4 ^{a}	−20.7±0.5 ^{a}	−20.4±0.6 ^{a}
DPR Korea	−25.6±0.4 ^{a}	−22.6±0.5 ^{a}	−21.2±0.5 ^{b}	−18.2±0.4 ^{a}	−22.8±0.3 ^{a}	−20.0±0.5 ^{a}
Korea	−23.3±1.6 ^{a}	−23.0±2.1 ^{a}	−22.7±2.2 ^{b}	−17.3±1.5 ^{a}	−22.8±2.0 ^{a}	−21.3±1.9 ^{a}

Table S6. Number of individual clams with geographical origins defined by LDA of multi-factor combinations. Numbers indicates the samples whose origin was well identified out of the samples from each country (China; n = 3, DPR Korea; n = 3, Korea; n = 51). The second panel represents cross-validation results for each geographical origin using leave-one-out cross-validation (LOOCV) method.

**Country**	**Number of correctly classified clams**			
	**SIA + FAs**	**Fas + δ^{13}C-FAs**	**SIA + δ^{13}C-FAs**	**All methods**
**China**	3	3	3	3
**Korea**	51	51	51	51
**DPR Korea**	3	3	3	3
**Total**	**57 (100%)**	**57 (100%)**	**57 (100%)**	**57 (100%)**
	Cross-validated value			
**China**	3	3	3	3
**Korea**	51	51	51	51
**DPR Korea**	2	3	3	3
**Total**	**56 (98.24%)**	**57 (100%)**	**57 (100%)**	**57 (100%)**
